# Feasibility and outcome of spleen and vessel preserving total pancreatectomy (SVPTP) in pancreatic malignancies — a retrospective cohort study

**DOI:** 10.1007/s00423-022-02690-7

**Published:** 2022-09-28

**Authors:** Christian Beltzer, Elio Jovine, Konstantin-Viktor Hesch, Derna Stifini, Laura Mastrangelo, Marco Huth, Alfred Königsrainer, Silvio Nadalin

**Affiliations:** 1 Department of General, Visceral and Transplant Surgery, University Hospital Tübingen, Tübingen, Germany; 2 Department of General, Visceral and Thoracic Surgery, Armed Forces Hospital Ulm, Ulm, Germany; 3 Department of Surgery, IRCCS AOU Sant’Orsola-Malpighi, Bologna, Italy; 4 Department of General and Visceral Surgery, Sigmaringen Hospital, Sigmaringen, Germany

**Keywords:** Total pancreatectomy, Vessel preservation, Spleen preservation, Postoperative pancreatic fistula, Splenectomy, Pancreatoduodenectomy

## Abstract

**Introduction:**

Total pancreatectomy (TP) is most commonly performed to avoid postoperative pancreatic fistula (POPF) in patients with high-risk pancreas or to achieve tumor-free resection margins. As part of TP, a simultaneous splenectomy is usually performed primarily for the reason of oncologic radicality. However, the benefit of a simultaneous splenectomy remains unclear. Likewise, the technical feasibility as well as the safety of spleen and vessel preserving total pancreatectomy in pancreatic malignancies has hardly been evaluated. Thus, the aims of the study were to evaluate the feasibility as well as the results of spleen and vessel preserving total pancreatectomy (SVPTP).

**Material and methods:**

Patient characteristics, technical feasibility, perioperative data, morbidity, and mortality as well as histopathological results after SVPTP, mainly for pancreatic malignancies, from patient cohorts of two European high-volume-centers for pancreatic surgery were retrospectively analyzed. Mortality was set as the primary outcome and morbidity (complications according to Clavien-Dindo) as the secondary outcome.

**Results:**

A SVPTP was performed in 92 patients, predominantly with pancreatic adenocarcinoma (78.3%). In all cases, the splenic vessels could be preserved. In 59 patients, the decision to total pancreatectomy was made intraoperatively. Among these, the most common reason for total pancreatectomy was risk of POPF (78%). The 30-day mortality was 2.2%. Major complications (≥ IIIb according to Clavien-Dindo) occurred in 18.5% within 30 postoperative days. There were no complications directly related to the spleen and vascular preservation procedure. A tumor-negative resection margin was achieved in 71.8%.

**Conclusion:**

We could demonstrate the technical feasibility and safety of SVPTP even in patients mainly with pancreatic malignancies. In addition to potential immunologic and oncologic advantages, we believe a major benefit of this procedure is preservation of gastric venous outflow. We consider SVPTP to be indicated in patients at high risk for POPF, in patients with multilocular IPMN, and in cases for extended intrapancreatic cancers.

## Introduction

Radical oncological resection with systematic lymphadenectomy is the only possible curative therapy for malignant pancreatic tumors [1]. Depending on the tumor localization, a pancreas head resection (pancreaticoduodenectomy, PD) or a resection of the left pancreas (distal pancreatectomy, DP) is usually performed. In particular cases (e.g., multilocular IPMN, to achieve tumor-free resection margins or to avoid POPF in patients with high fistula risk score/FRS [2]), extension of the procedure to total pancreatectomy (TP) may be required [3–5].

The role of simultaneous splenectomy in the context of TP is still debated. For DP and TP, both the “National Comprehensive Cancer Network (NCCN)” and the “European Society for Medical Oncology (ESMO)” guidelines [6, 7] define simultaneous splenectomy as the surgical standard. The reasons for recommending simultaneous splenectomy are technical (i.e., the close anatomical relation of the pancreas to the splenic artery and vein, which makes it difficult to preserve the vessels supplying the spleen) and oncological ones (i.e., avoidance of any preparation or dissection near the tumor in the sense of a “no-touch-technique”). Similarly, the “International Study Group for Pancreatic Surgery (ISGPS)” recommends simultaneous splenectomy for carcinoma of the pancreatic body or tail in their consensus statement from 2014 [8]. However, there is no level-1-evidence regarding this issue, and an oncological benefit of simultaneous splenectomy has scarcely been investigated and demonstrated.

On the other hand, spleen preservation has been suggested to have potential favorable implications in terms of (1) immunologic, (2) hematologic, (3) hemodynamic, and (4) oncologic outcome [9–15].

Previous publications on spleen preservation in pancreatic resections are mostly limited to the following situations: (1) spleen preservation within the scope of an (extended) DP [10, 11, 14, 16] and TP for benign or “low grade” malignant diseases [17].

In most of the reported cases of spleen preserving DP, the splenic vessels are being resected according the “Warshaw-Procedure” [18, 19] (i.e., central ligation of the splenic vessels with maintenance of the splenic perfusion via short vessels from the large gastric curvature).

In contrast to that, Jovine et al. reported for the first time the technique of spleen and vessel preserving TP (SVPTP) in 2004 [20], and Kimura for DP in 2010 [16]. Although advantages of spleen preservation can be assumed as mentioned above, the procedure has hardly been followed up to now. There is only one case report on spleen and splenic vessel preservation in the context of a TP for pancreatic cancer [21].

The aims of our retrospective analysis were to investigate feasibility, safety, and postoperative outcomes of SVPTP in 92 patients from two high-volume centers.

## Material and methods

We retrospectively analyzed our bi-centric experience with all patients undergoing SVPTP. Patient characteristics (gender; age; preoperative diagnosis; American Society of Anesthesiologists-score, ASA; body-mass-index, BMI; comorbidities; neoadjuvant chemotherapy or radiation; pre-existing insulin-dependent diabetes mellitus, IDDM) and operative parameters (reason to switch from PD or DP to TP, vascular reconstruction) were collected. Mortality was set as the primary outcome and morbidity as the secondary outcome. Postoperative complications were classified according to the Clavien-Dindo classification [22]. The recording of postoperative complications was limited to a period of 90 days. Parameters were described in absolute values as well as mean value and range. Oncological parameters were assessed based on tumor entity and stage, tumor localization (head, body, tail), and postoperative histopathological findings.

This retrospective study has been carried out in accordance with the Code of Ethics of the World Medical Association (Declaration of Helsinki). Ethics approval was given by European Commission (ethics approval number: CE 19,163) as well as the Ethics Committee of the University of Tübingen (ethics approval number: 260/2022BO2).

### Surgical procedure sequence of SVPTP

**Fig. 1:**
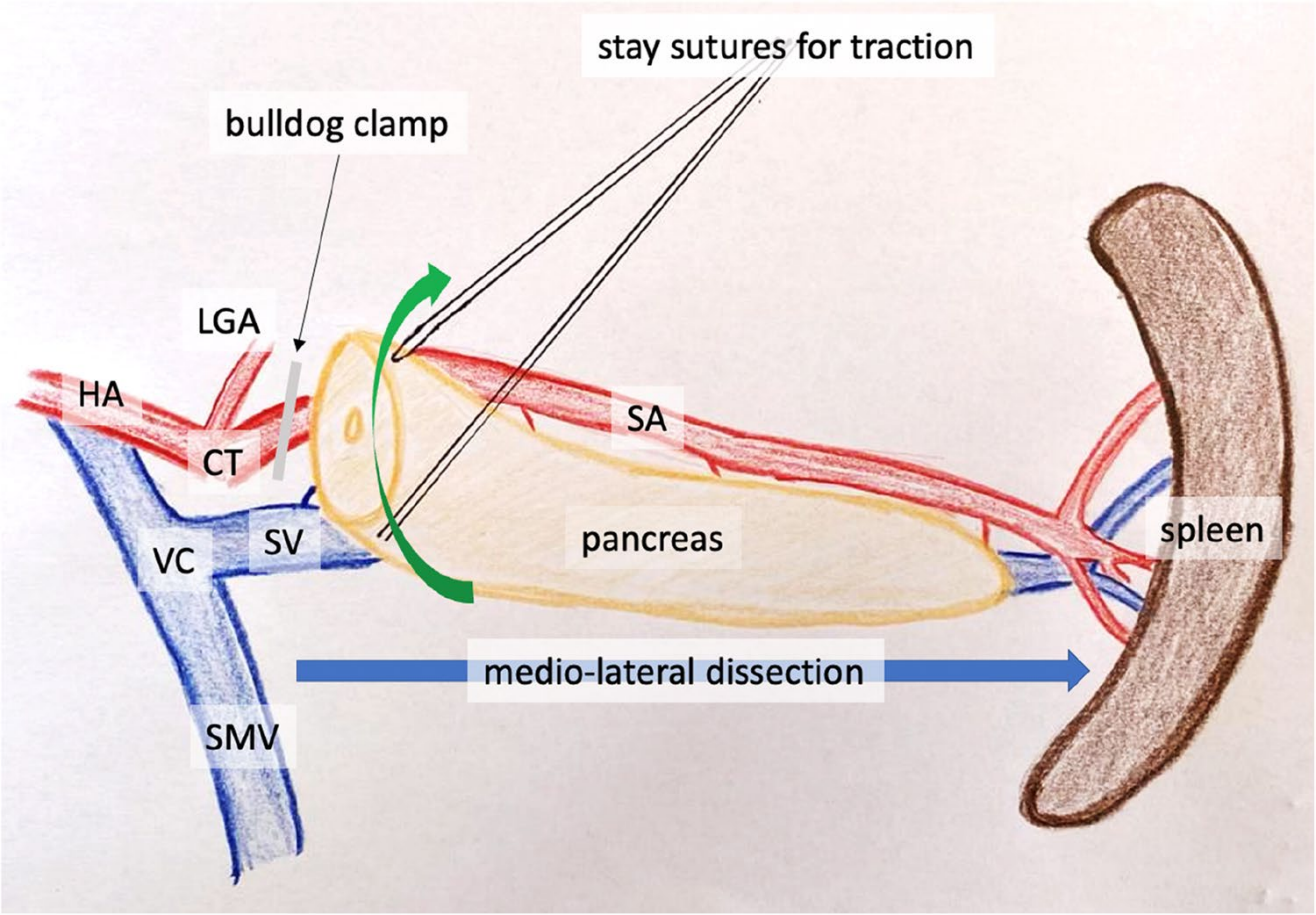
Illustration of surgical sequence of SVPTP (head of pancreas already resected), bulldog clamp on splenic artery to reduce venous return, stay sutures to lift pancreas from the splenic vein; CT, celiac trunk; HA, hepatic artery; LGA, left gastric artery; PV, portal vein; SA, splenic artery; SV, splenic vein; SMA, superior mesenteric artery; SMV, superior mesenteric vein; SVPTP, spleen and vessel preserving total pancreatectomy; VC, venous confluens

**Fig. 2:**
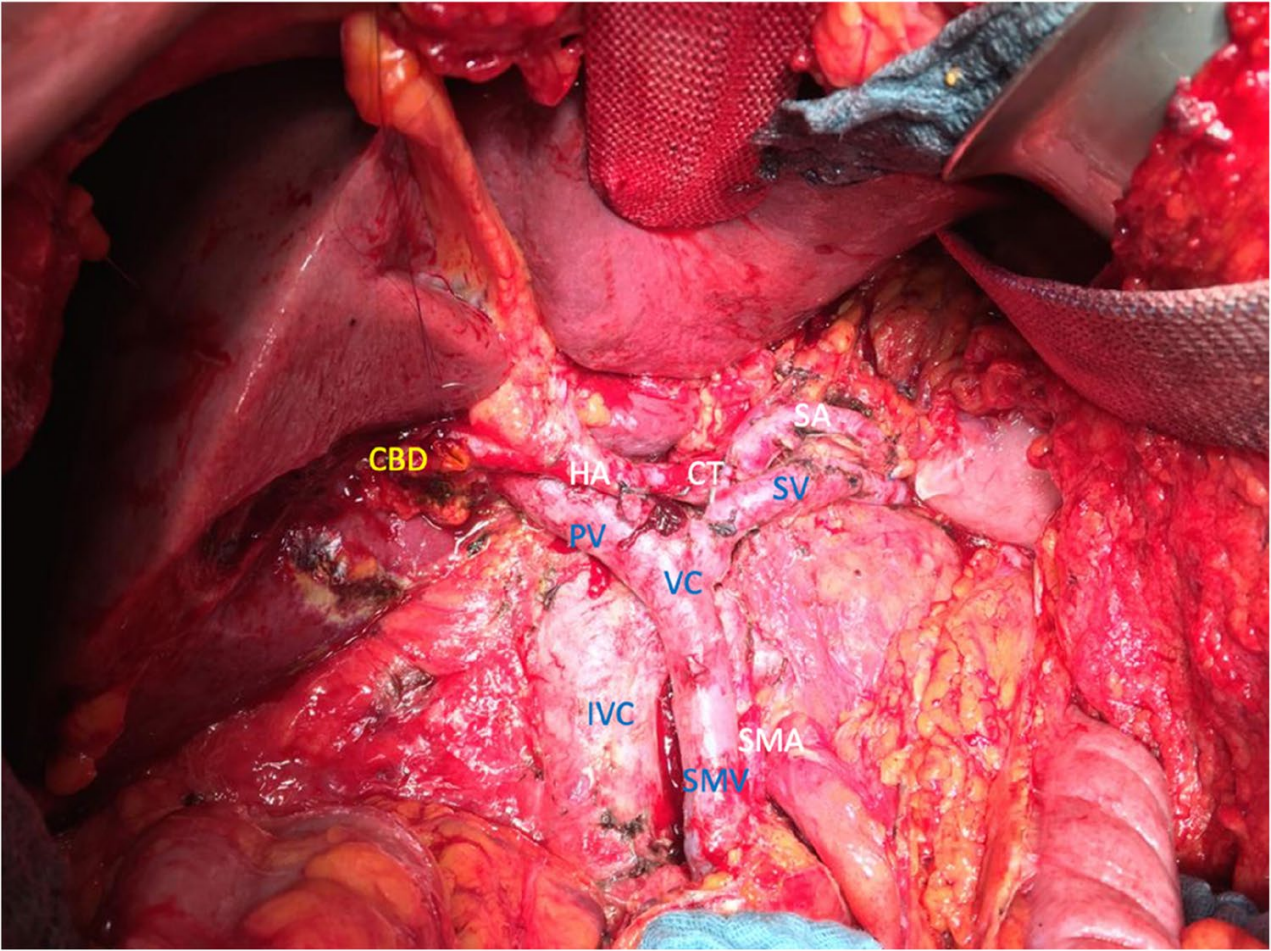
Anatomical structures after SVPTP; CBD, common bile duct; CT, celiac trunk; HA, hepatic artery; IVC, inferior vena cava; PV, portal vein; SA, splenic artery; SV, splenic vein; SMA, superior mesenteric artery; SMV, superior mesenteric vein; SVPTP, spleen and vessel preserving total pancreatectomy; VC, venous confluens

Regardless of the preoperative planned resection (PD, DP, TP), the pancreas head was first divided from the body at the level of the pancreatic neck. The pancreas was always transected providing an adequate macroscopic distance from the tumor, in order to avoid tumor cell dissemination. Division of the pancreas firstly provided a good exposure of the vascular anatomy (PV, superior mesenteric vein/SMV, venous confluens/VC) and secondly facilitated further vessel-preserving dissection in medio-lateral direction towards the spleen. The splenic artery was temporarily clamped to minimize venous return and pressure in the SV, thus reducing bleeding from side branches of the SV (Figs. 1 and 2). The side branches of the splenic artery and vein were selectively divided between sutures or clips. The short gastric vessels between the spleen and the great gastric curvature were preserved to maintain additional gastric blood flow. A standardized lymph node dissection was performed along the hepatoduodenal ligament, hepatic artery, celiac trunk, right side of the superior mesenteric artery (SMA), and the splenic artery to the splenic hilus. Duodenum, first jejunal loop, and common bile duct (CBD) were resected “en-bloc” with the head of the pancreas. In case of infiltration of the PV or of the VC, a partial vascular resection and reconstruction were performed (Tables 1, 2 and 3). Thus, even in cases of venous tumor infiltration, the splenic vein could be preserved.

**Table 1:** Patient demographics and preoperative conditions; all patients included (*n* = 92)

Characteristics	Value
Gender (*n* [%])	
Male	48 [52.2]
Female	44 [47.8]
Age (mean ± range)	67.3 (± 12.2)
BMI (mean ± range) ACCI (mean)	25.2 (± 4.5) 5.2
ASA-score (mean)	2.7
Karnofsky index (mean, [%])	[86.8]
ECOG performance status (mean)	0.29
Diabetes (*n* [%])	37 [40.2]
IDDM	19 [20.7]
NIDDM	18 [19.6]
Preoperative diagnosis* (*n* [%])	
PDAC	81 [88.0]
NET	5 [5.4]
CCA	3 [3.3]
IPMN	2 [2.2]
Pancreatitis	1 [1.1]
Preoperative biliary drainage (*n* [%]) ERCP PTDC	27 [29.3] 23 [25] 4 [4.3]
Preoperative chemotherapy (*n* [%])	4 [4.3]
Preoperative radiation (*n* [%])	2 [2.2]

*ACCI*, age-adjusted charlson comorbidity index score; *ASA*, American Society of Anesthesiologists; *BMI*, body-mass-index; *CCA*, cholangiocarcinoma; *ECOG*, Eastern Cooperative Oncology Group; *ERCP*, endoscopic retrograde cholangiopancreaticography; *IDDM*, insulin dependent diabetes mellitus, *IPMN*, intraductal papillary mucinous neoplasm; *NET*, neuroendocrine tumor; *NIDDM*, non-insulin dependent diabetes mellitus; *PDAC*, pancreatic ductal adenocarcinoma; *PTCD*, percutaneous transhepatic cholangiography and drainage

*Preoperative diagnosis predominantly based on imaging

**Table 2:** Reconstructed vessels and types of vascular reconstructions performed (*n* = 20)

Vessel	Type of vascular resection and reconstruction	Value (*n* [%])
SMV	Segmental venous resection; end to end anastomosis	3 [15]
SMV + SV	Segmental venous resection; end to end anastomosis	1 [5]
SMV + PV + SV	Segmental venous resection; end to end anastomosis	1 [5]
SMV + PV + IMV	Segmental venous resection; end to end anastomosis with interposition graft	1 [5]
PV	Segmental venous resection; end to end anastomosis Tangential venous resection; including 1 patch	7 [35] 5 [25]
PV + SV	Segmental venous resection; end to end anastomosis	2 [10]

*IMV*, inferior mesenteric vein; *PV*, portal vein; *SMV*, superior mesenteric vein; *SV*, splenic vein

**Table 3:** Pancreatic diseases and histological entities, all patients included (*n* = 92)

Pancreatic disease and histological entity	Value (*n* [%])
Malignant diseases PDAC NET Metastatic tumor Breast cancer metastases Renal cell carcinoma metastases CCA	85 [92.4] 72 [78.3] 5 [5.4] 5 [5.4] 1 [1.1] 4 [4.3] 3 [3.3]
Non-malignant diseases IPMN Pancreatitis	7 [7.6] 5 [5.4] 2 [2.2]

*dCCA*, distal cholangiocarcinoma; *iCCA*, intrahepatic cholangiocarcinoma; *pCCA*, perihilar cholangiocarcinoma; *IPMN*, intraductal papillary mucinous neoplasm; *NET*, neuroendocrine tumor; *PDAC*, pancreatic ductal adenocarcinoma

## Results

A total of 92 patients were treated with SVPTP in two European high-volume centers for pancreatic surgery.

### Patient characteristics

The mean age of the patient was 67.3 years, the mean BMI was 25.2, and 19 patients (20.7%) had a pre-existing insulin-dependent diabetes mellitus (IDDM). The mean ACCI (age-adjusted Charlson comorbidity index score) was 5.2, the mean Karnofsky index was 86.6 points, and the mean ECOG (Eastern Cooperative Oncology Group) performance status was 0.26. Preoperative diagnoses were made mainly on the basis of imaging. Among them, PDAC was the most common preoperative diagnosis in 81/92 patients (88.0%). Preoperative biliary drainage was performed in 27 patients (29.3%), and 4 patients (4.3%) received preoperative chemotherapy. Details on patient characteristics are provided in Table 1.

### Indications for SVPTP

Out of a total of 92 patients, pancreatic resections were planned as PD (*n* = 58), TP (*n* = 33), and DP (*n* = 1) preoperatively. From 58 resections planned as PD, the most common indication for intraoperative switching to TP was patients at risk for a postoperative pancreatic fistula (POPF; *n* = 46, 78%), mainly based on a small pancreatic duct diameter (3 mm: *n* = 32; 2 mm: *n* = 14) and a soft gland texture (*n* = 46). Among these, the average fistula risk score (FRS) [2] was 4.4 points (4 points: *n* = 30; 5 points: *n* = 13; 6 points: *n* = 3). In 20 patients (34%; PD: *n* = 19; DP: *n* = 1), repeated tumor-positive frozen sections of the resection margin (R1 resection margin) were another indication to extend the resection from PD or DP to TP.

In cases of an assessed high risk for POPF, concomitant vascular reconstruction (*n* = 20) or pre-existing IDDM facilitated the decision to perform total pancreatectomy.

### Vascular resections and reconstructions

In 20 cases (21.7%), a partial resection and reconstruction of the portal vein (PV), superior mesenteric vein (SMV), or splenic vein (SV) were performed. In most cases, these were segmental resections with end-to-end anastomosis (Table 2, Fig. 2). No arterial vascular resections were performed.

### Histopathological results, tumor localization, and staging (see Table 3)

Postoperative histopathological results revealed a malignant disease in the majority of patients (*n* = 85, 92.4%), among which ductal adenocarcinomas (PDAC) accounted for the largest proportion (*n* = 72, 78.3%). Other malignant entities included neuroendocrine tumors (NET; *n* = 5, 5.4%), extrahepatic cholangiocarcinomas (CCA; *n* = 3, 3.3%), and metastases from breast and renal cell carcinomas (*n* = 5, 5.4%). Seven out of 92 patients (7.6%) had nonmalignant disease (5 IPMN and 2 pancreatitis, Table 3).

Tumors were predominantly located in the head and neck of the pancreas (*n* = 76, 90.5%), 4 tumors (4.8%) affected the whole pancreas, and only 4 (4.8%) were carcinomas of the left pancreas. Among 85 patients with malignant disease, 61 (71.8%) had tumor-free resection margins on histologic examination. The mean number of harvested lymph nodes was 22 (with a range from 10 to 50). Vascular infiltration by the tumor was confirmed histologically in 10 out of 20 patients (50%) with vascular resection.

Tumor stage of all patients with PDAC was classified according to the American Joint Committee on Cancer (AJCC) system, 8th edition [23]. Among them were predominantly patients with nodal positive tumors (tumor stage ≥ IIB = 72.3%; Table 4).

**Table 4:** Staging of patients with PDAC (*n* = 72) according to *AJCC*, American Joint Committee on Cancer; *PDAC*, pancreatic ductal adenocarcinoma; *IA*, T1 N0 M0; *IB*, T2 N0 M0; *IIA*, T3 N0 M0; *IIB*, T1-3 N1 M0; *III*, T4 any N M0; *IV*, any T any N M1

Tumor stage PDAC (according to AJCC)	Value (*n* [%])
IA	4 [5.6]
IB IIA IIB III IV	8 [11.1] 8 [11.1] 38 [52.8] 12 [16.7] 2 [2.8]

### Postoperative morbidity and mortality

Major complications (Clavien-Dindo ≥ IIIb–V) occurred in 17 patients (18.5%) within 30 days, and in 5 more patients (*n* = 22, 23%) within 90 days after SVPTP. Reoperation (Clavien-Dindo IIIb) was required in 9 patients (9.8%). Indications for reoperation were hemoperitoneum (*n* = 4), bile leakage (*n* = 1), burst abdomen (*n* = 1), abdominal sepsis (*n* = 1), gastric perforation (*n* = 1), and leakage of gastro-jejunal anastomosis.

Two patients (2.2%) died within 30 days after SVPTP, including 1 patient with acute stroke on POD 10, and 1 patient with multiorgan failure due to sepsis on POD 15. Another 3 patients (5.4%) died within 90 days after SVPTP (Table 5). Of these, 2 patients died of pneumogenic sepsis (POD 53 and 82), and 1 patient died cancer related (PDAC) on POD 90. No death was directly related to the SVPTP procedure. No case of gastric venous congestion was observed.

**Table 5:** Complications according to Clavien-Dindo

Grade of complication	0–30 POD, value (*n* [%])	0–90 POD, value (*n* [%])
I	4 [4.3]	4 [4.3]
II	52 [56.5]	53 [57.6]
IIIa	9 [9.8]	9 [9.8]
IIIb	9 [9.8]	9 [9.8]
IVa	6 [6.5]	7 [7.6]
IVb V	0 [0] 2 [2.2]	1 [1.1] 5 [5.4]

*POD*, postoperative day; grades: *I*, any deviation from normal postoperative course without treatment or intervention; *II*, pharmacological treatment or blood products; *IIIa*, intervention not under general anesthesia; *IIIb*, intervention under general anesthesia; *IVa*, life-threatening complication with single organ failure; *IVb*, life-threatening complication with multiorgan dysfunction; *V*, death of patient

## Discussion

The role of SVPTP in pancreatic surgery and especially for malignant tumors has been unexplored up to now. Based on our experience, which is to our knowledge the largest case series worldwide in pancreatic cancer, we were able to demonstrate that SVPTP is technically challenging but feasible and associated with low mortality rates.

In primarily planned partial resection of the pancreas (PD or DP), a TP can be indicated to for surgical radicality (i.e., positive resection margin, vascular infiltration, multilocular tumors, or premalignant lesions) or to avoid POPF, especially in patients with high FRS [24–27]. Despite different surgical techniques of pancreatic anastomosis, POPF remains a relevant problem in pancreatic surgery. In turn, postpancreatectomy hemorrhage (PPH) is the most serious consequence of POPF and is associated with high mortality. Patients with vascular resection and reconstruction might be particularly at risk of PPH in case of POPF. Stoop et al. demonstrated in a recent study that TP even in the consequence of type III DM (pancreoprivic diabetes mellitus) is equivalent to PD in long-term surgical outcome and quality of life [28].

Once the decision to perform a TP has been taken, the next question which arises is with or without spleen preservation. It is obvious that TP with splenectomy is easier to perform than spleen preservation. We believe, however, that *surgical feasibility* is a weak or inappropriate rationale for performing splenectomy.

To adequately perform SVPTP, based on our experience, we suggest to first transect the pancreas at the level of the pancreatic neck. This facilitates exposure and dissection of the splenic vessels. Of course, if the tumor is located in the region of the pancreatic neck, the pancreas should be transected at a safe distance from the tumor. In rare cases, it may also be necessary to remove the entire pancreas “en bloc” without prior transection, which, however, makes the performance of SVPTP technically much more difficult.

Furthermore, *oncological aspects* can be cited as a justification for simultaneous splenectomy. In our view, simultaneous splenectomy should always be performed in cases of direct tumor infiltration of the splenic vessels or the spleen itself. In such cases, splenic preservation is not feasible and reasonable. However, an indication for an obligatory simultaneous splenectomy as part of a TP in malignant pancreatic malignancies cannot be derived from the available data. Collard et al. retrospectively examined the frequency of nodal metastasis in the hilum of the spleen (lymph node station 10) in left PDAC [29]. A total of 104 patients were included in the analysis; 40% had a nodal positive tumor stage but neither a node metastasis in lymph node station 10. Consequently, the oncological sense of a supposedly better lymph node radicality at the splenic hilum with an obligatory simultaneous splenectomy is questioned. Another retrospective histologic study after distal pancreatectomy with inclusion of 130 specimens (85 adenocarcinomas, 37 NET and 8 other carcinomas; 59 corpus and 71 tail carcinomas) reached a similar conclusion. In only one case of tail carcinoma, a lymph node metastasis was found close to the splenic hilum [30]. Kim et al. also found no lymph node metastasis at the splenic hilum in their study of 12 patients with radical antegrade pancreatosplenectomy for PDAC [31]. Furthermore, it is essential to consider, that splenectomy may be also related to a generally increased risk of cancer development, as shown in a recent observational study (hazard ratio for cancer development in non-traumatic splenectomy = 2.64) [13]. Animal experimental data indicate a possible negative influence of splenectomy on the oncological prognosis: in mice with induced pancreatic carcinoma, tumor progression in groups with and without splenectomy was compared. Following splenectomy, both local tumor growth in the pancreas tail and the incidence of peritoneal carcinomatosis were significantly increased compared to the group without splenectomy [12]. Whether spleen preservation has an influence on the long-term oncological outcome in humans remains unclear to date. Even with our study, this question cannot be answered because of the retrospective design and the lacking long-term results.

Splenectomy seems also to be associated with immunological and/or hematological complications [10, 11, 32–34]. Elevated platelet count has been shown after splenectomy in comparison to spleen-preserving procedures [14]. Tezuka et al. showed that splenic preservation during distal pancreatectomy was associated with a more rapid and long-term normalization of white blood cell count (WBC) and platelet count [15]. However, there may be only a low risk of thromboembolic complications in cases of thrombocytosis [35].

Once the decision is made to perform a TP with spleen preservation, the next question is *how to perform it technically*, Warshaw-Procedure or SVPTP.

The technically simpler WP for spleen-preservation is associated with higher rates of postoperative splenic infarction, which, however, only partially requires a secondary splenectomy [10, 36]. In addition, the venous outflow of the spleen exclusively via the large gastric curvature causes gastric varices after WP [36]. A meta-analysis published in 2019 comparing WP and SVPDP [37] showed a significantly higher rate of gastric varices after WP.

The main advantage of SVPTP results in the maintenance of the physiological portal venous flow (even at costs of reconstruction and reimplantation of splenic vein in the portal vein), which theoretically could have a preventive effect on the development of portal vein thrombosis. After resection of the splenic vein, left-sided portal hypertension (LSPH) may occur. Relevant venous collaterals after central ligation of the splenic vein are (1) left gastric vein, (2) middle colonic vein, and (3) superior right colic vein arcade [38]. Left-sided portal hypertension may in turn result in varices in the stomach and colon [9], which is caused by impaired venous return from the inferior mesenteric vein and the left gastric vein [39]. Ono et al. used intraoperative splenic vein pressure measurement before and after clamping to predict left-sided portal hypertension [40]. However, if the splenic vein cannot be preserved, reconstruction or reanastomosis of the splenic vein should always be attempted. In addition to the portal vein, the left renal vein is also suitable for reconstruction [41].

To reach a conclusion about the *safety of SVPTP*, our own findings must be compared with those of TP with simultaneous splenectomy. Major complications (Clavien-Dindo ≥ IIIb) occurred in 23% within 90 days after surgery, and 90-day mortality was 5.4% in our series of 92 SVPTP patients. TP is known to be associated with high overall perioperative morbidity, with reported major complications of 25.5–34.5% and a mortality of 4–23% [28, 42–47]. In this regard, our own results are in agreement with the data in the literature on TP. In this context, Loos et al. [47] recently demonstrated that life-threatening gastric venous congestion (GVC) occurs in 27% in association with TP, which in turn necessitates partial or total gastrectomy. They demonstrated that both splenectomy and resection of the coronary vein are associated with a significantly increased risk of GVC. In our series, no gastric venous congestion occurred.

The preoperative general condition of patients may also have a relevant impact on surgical outcome. Based on the ACCI, Karnofsky index, and ECOG, patients in our cohort (mean AACCIS: 5.2; ECOG: 0.29; Karnofsky index: 86.8) could be classified as suitable for surgery [48–50].

In summary, based on our results, SVPTP can be considered safe for two main reasons:

1. There are no notable differences with respect to morbidity and mortality when comparing our own results of SVPTP with the data of TP with simultaneous splenectomy in the literature.
2. No complications attributable to SVPTP specifically were observed in our patients.

In addition, from hemodynamic aspects, we consider SVPTP to be even safer than TP with splenectomy. In SVPTP, the short gastric and the left gastroepiploic vessels are preserved. In TP with simultaneous splenectomy, the arterial perfusion of the stomach is provided exclusively via the left gastric artery. Particularly, in the case of stenosis of the celiac trunk, preservation of the splenic artery becomes crucial, as otherwise, a gastrectomy would be necessary. The same is the case for the venous outflow of the stomach to the splenic vein via the short gastric vessels, which is maintained in SVPTP. Thus, even in event of resection of the coronary vein, venous outflow is preserved and a life-threatening GVC can be omitted.

WP should therefore be reserved for cases of direct tumor infiltration of the splenic vessels or the spleen. In all other cases, the aim should always be to maintain the physiological blood supply to the spleen, and indirectly to the stomach, and thus to achieve SVPTP.

When interpreting the results presented here, the retrospective study design and lacking data concerning oncological follow-up (overall survival/OS; disease free survival/DFS) are the main limiting factors. Therefore, oncological conclusions cannot be derived from our study.

## Conclusion

Based on our experience and the mentioned results of our patients undergoing SVPTP, we were able to demonstrate that the procedure is technically feasible but challenging and associated with low morbidity and mortality rates.

Advantages of SVPTP are maintenance of arterial and venous perfusion of the stomach, and thus avoidance of gastric venous congestion, potential hematological, immunological, and hemaodynamic advantages, as well as avoidance of left-sided portal hypertension. In our view, TP performed either to avoid POPF in patients with high FRS or to achieve a tumor-free resection margin should always be performed as SVPTP — provided that there is no direct tumor infiltration of the splenic vessels or the spleen itself.

Evidence for or against simultaneous splenectomy in the setting of TP with focus on pancreatic malignancies could only be derived from a randomized comparative study. Since such a study has not yet been conducted, recommendations can therefore only be formulated cautiously.

## Explanations

ACCI (Age adjusted Charlson Comorbidity Index Score): diseases with score 1, myocardial infarction, congestive heart failure, peripheral vascular disease, dementia, cerebrovascular disease, chronic pulmonary disease, ulcer disease, diabetes, hypertension, mild liver disease; score 2, moderate or severe renal disease, hemiplegia, malignant lymphoma, any tumor; score 3, moderate or severe liver disease; score 6, metastatic solid tumor, acquired immune deficiency syndrome; score 1 for age for each decade over age 40 years (up to 4 points).

ECOG (Eastern Cooperative Oncology Group): grade 0, fully active; grade 1, restricted in physically strenuous activity but ambulatory and able to carry out work of a light or sedentary nature; grade 2, ambulatory and capable of all selfcare but unable to carry out any work activities; grade 3, capable of only limited selfcare; confined to bed or chair more than 50% of waking hours; grade 4, completely disabled, cannot carry on any selfcare, totally confined to bed or chair; grade 5, dead.

Karnofsky index: 100, normal, no complaints, no evidence of disease; 90, able to carry on normal activity, minor signs or symptoms of disease; 80, normal activity with effort, some signs or symptoms of disease; 70, cases for self but unable to carry on normal activity or to do active work; 60, requires occasional assistance but is able to care for most of personal needs; 50, requires considerable assistance and frequent medical care; 40, disabled, requires special care and assistance; 30, severely disabled, hospitalization is indicated although death not imminent; 20, very ill, hospitalization and active supportive care necessary; 10, moribund; 0, dead.
